# Using Match Reference Values of External Load to Monitor Performance and Fatigue in Football: Are We Looking the Right Way?

**DOI:** 10.1186/s40798-026-01112-y

**Published:** 2026-09-20

**Authors:** Ricardo Pimenta, Hugo Antunes, José Afonso, Fábio Yuzo Nakamura

**Affiliations:** 1 Research Center in Sports Sciences, Health Sciences and Human Development (CIDESD), University of Maia, 4475-690 Maia, Portugal; 2 Research Center of the Polytechnic Institute of Maia (N2i), Maia Polytechnic Institute (IPMAIA), Castêlo da Maia, 4475-690 Maia, Portugal; 3 Department of Rehabilitation and Optimization of Performance (DROP), Futebol Clube de Famalicão - Futebol SAD, Famalicão, Portugal; 4 FSI Lab, Football Science Institute, Granada, Spain; 5 Department of Neurosciences, Biomedicine and Movement Sciences, University of Verona, 37124 Verona, Italy; 6 Centre for Research, Education, Innovation and Intervention in Sport (CIFI2D), Faculty of Sport of the University of Porto, Rua Dr. Plácido Costa, 91, 4200-450 Porto, Portugal; 7 Graduate Program in Physical Education, Federal University of Pernambuco, Recife, Brazil

**Keywords:** Soccer, Microcycle management, Load variability, Training prescription, Fatigue monitoring

## Abstract

Monitoring external load is critical for informed decision-making processes across sports. In football, external load is routinely monitored during training sessions and competitive matches. Matches are typically given particular emphasis, as they are generally associated with the highest external load and the most pronounced fatigue responses within a microcycle. Consequently, coaches frequently use match-derived external load values as reference points for prescribing training loads throughout this period. However, despite the apparent specificity of this approach and the unique characteristics of match external load, recent research suggests that coaching staffs may be inadequately quantifying the actual external load produced by players during matches, as well as the associated fatigue responses. This limitation is largely attributable to the substantial match-to-match variability observed in external load outcomes, which may lead to misinterpretation of the true load produced by players. Therefore, a normalized approach to external load monitoring, whereby locomotor external loads are expressed to each player’s highest observed load, has been recommended as a more player-specific method for quantifying and interpreting external load in football. This opinion article highlights how the use of match load as a reference for training prescription may lead to inaccurate estimations of the external load imposed on football players. Furthermore, it outlines the potential consequences of match-load-based prescription for microcycle load management, which may ultimately result in suboptimal or ineffective training stimuli in the long term. Finally, we propose a conceptual framework in which external locomotor load metrics are expressed to players’ characteristic- and/or exposure-based reference values. This framework is intended to support a more player-specific interpretation of external load but requires empirical validation before being used as a prescriptive decision-making model.

## Key Points

- The widespread practice of using match-derived external load as a reference for training prescription in football may be limited by the substantial variability and context-dependency of match loads.
- Consequently, this approach may lead to misinterpretation of players’ true external load and fatigue responses and, in turn, to suboptimal training stimuli.
- A shift toward normalized external load metrics using individual characteristic- and exposure-based references may provide a more personalized framework for external load monitoring and training prescription in football.

## Introduction

Training load management consists of strategically manipulating training frequency, volume, and intensity across the competitive season to ensure a sufficient stimulus for positive adaptation while hopefully minimizing injury risk [1, 2]. Its practical implementation is often limited by methodological constraints, particularly the reliance on the match as a reference for structuring the subsequent microcycle (i.e., a reactive approach) and the use of fixed speed and/or acceleration thresholds derived from Global Navigation Satellite Systems (GNSS) to quantify external load. In football, microcycles are commonly structured according to the temporal proximity of each session to match day (MD), using the match day minus/plus (MD ±) format [3]. Within this framework, sessions are classified based on their primary objective: post-match recovery (MD+X) or preparation for the subsequent match (MD−X). The “+X” and “−X” designations indicate the number of days after or before the match, respectively [4]. In a one-match-per-week schedule, the microcycle typically follows the sequence: MD+1, MD−4, MD−3, MD−2, and MD−1 [5].

In football, external load is typically quantified using locomotor variables, such as high-intensity accelerations (ACC) and decelerations (DEC), absolute high-speed running distance (HSR_ABS_), absolute sprint distance (Sprint_ABS_), and total distance (TD) [3, 6]. Using absolute thresholds during a 90-min match, football players typically cover an average 618–1001 m of high-speed running (HSR_ABS_, distance between 19.8 km/h and 25.2 km/h) [6], 153–295 m of sprinting distance (Sprint_ABS_, distance ≥ 25.2 km/h) [6], and perform 19–61 high-intensity accelerations (ACC_ABS_) and 25–58 decelerations (DEC_ABS_) [7, 8]. Notably, the absolute values of these metrics are dependent on the thresholds applied to define high-intensity actions, and there is no single, universally accepted set of thresholds. Nonetheless, these values are higher than those usually recorded in training sessions, where players have been reported to train for an average of 65–77 min, covering 41–205 m of HSR_ABS_ [1, 8–11].

**Fig. 1:**
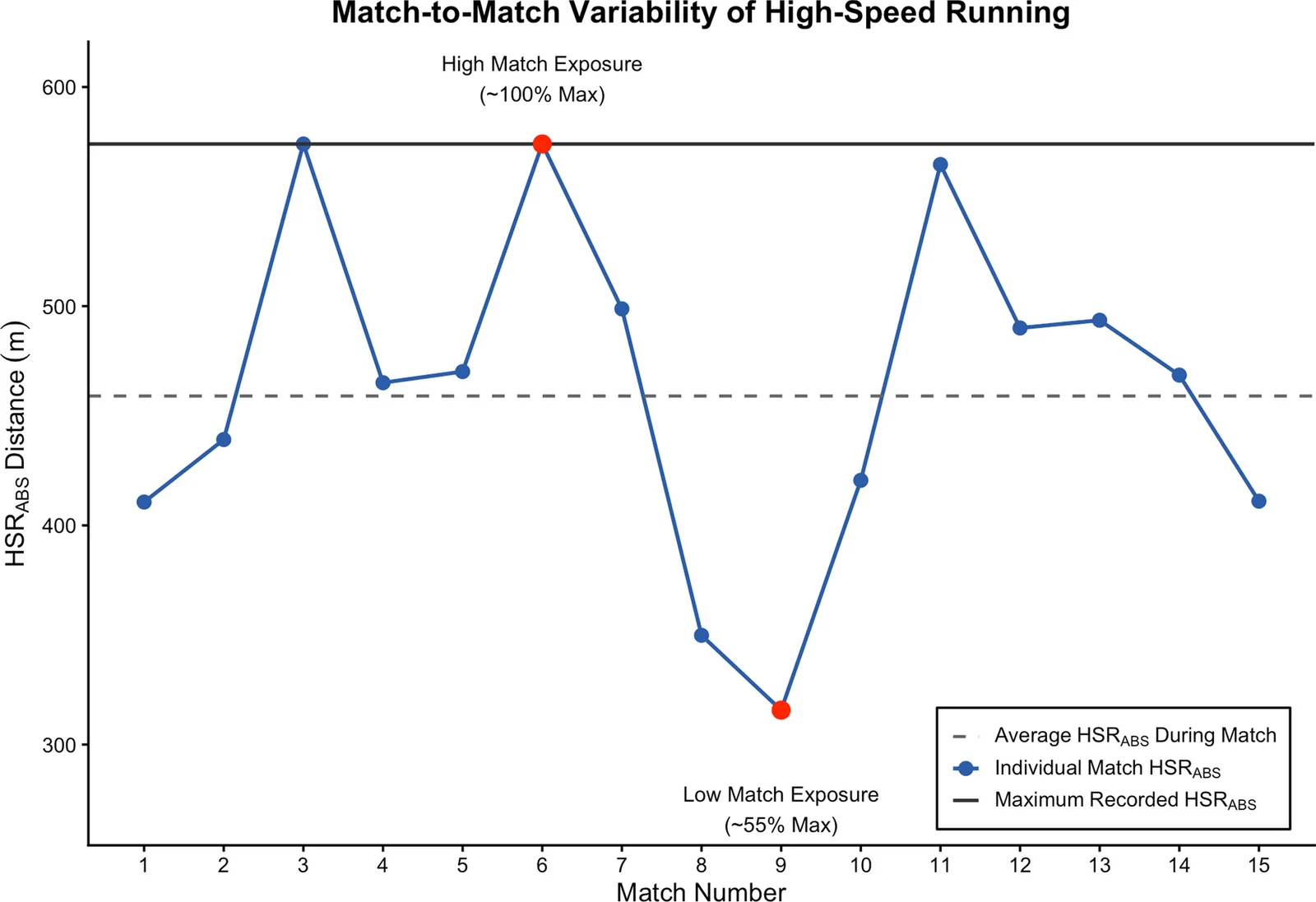
Variability of the absolute high-speed running (HSR_ABS_) distance covered by a center back. HSR_ABS_, absolute high-speed running distance. Adapted from Gregson et al. (2010)

Previous research has highlighted substantial match-to-match variability (considering only players completing entire matches) in high-intensity locomotor metrics, including HSR_ABS_ (CV = 20.6–29.8%) [12], ACC_ABS_ (CV = 26.9%), and DEC_ABS_ (CV = 27.9%) [13]. For instance, a player with an average HSR_ABS_ of ~459 m per match could experience fluctuations associated with a CV of 18.8%; if the player’s maximal HSR_ABS_ is 574 m, in one match he may reach nearly 100% of this maximum, while another match elicits only ~55% [14] (Fig. 1).

Match-to-match variability in external load has been associated with contextual factors, such as playing position, style of play, and the competitive level of the opposition, underscoring the need for a thorough consideration regarding the most appropriate reference value for external load prescription and monitoring. Moreover, relying on absolute-threshold-based metrics can substantially misrepresent the locomotor intensity represented by the external load performed [15–18]. Accordingly, recent evidence supports the normalization of locomotor metrics, which anchors thresholds to player-specific reference values rather than generalized cutoffs, which may provide a more player-specific representation of external load exposure [15–18]. This opinion article aims to: (1) discuss the rationale for using the highest individual observed load as an alternative exposure-based reference to the previous match or maximum match loads and (2) examine the conceptual basis for using normalized thresholds to the player’s characteristics (such as maximum speed) rather than absolute thresholds.

## Methodological Framework

In a typical football microcycle, external training load is adjusted according to factors, such as match-induced fatigue [19], the outcome of the previous match [20], or the quality of subsequent opponents [20]. Coaching staff commonly use match load as a reference to structure training [8, 19, 21], often prescribing sessions as a percentage of the previous match load [22]. Although MD is generally considered the highest-load session of the microcycle [23–25], its magnitude requires a nuanced interpretation. The relative cumulative load for each locomotor metric in each session of the microcycle is calculated as follows:

$$Percentage\, Load (\%) = (\frac{Average\, Value\, of\, the\, Metric\, on\, MD\pm }{Average\, Value \,of\, the\, Metric\, on\, MD or\, Maximum})* 100$$

where MD± represents the specific session of the microcycle relative to MD.

Match load can be conceptualized either as (i) the load generated in the previous match or (ii) the highest match load recorded across the season. Both approaches are limited by match-to-match variability and positional differences, preventing the establishment of a stable and representative reference of the player’s highest observed external load. This highlights the need for an alternative exposure-based reference that considers the player’s highest external load observed across both training and match contexts. Training provides a higher frequency of observations than matches, increasing the likelihood of capturing typical performance profiles and outputs exceeding match-derived reference values (i.e., relative to match performance), particularly relevant for non-starters and players who do not complete full matches. Moreover, emerging evidence further indicates that the highest locomotor loads are not always achieved in competition as superior values could occur in contexts, such as structured testing [26] or specific training drills [27].

Training environments provide relatively more structured and regulated conditions than matches, to some extent limiting the variability in external loads. Conversely, match-play shows pronounced match-to-match variability in high-intensity locomotor metrics.

In addition, match load is typically quantified using absolute thresholds, which could underestimate true locomotor external load for most players [15–17]. Since players do not always express their highest locomotor outputs during matches, maximum values could instead occur in non-competitive contexts.

**Fig. 2:**
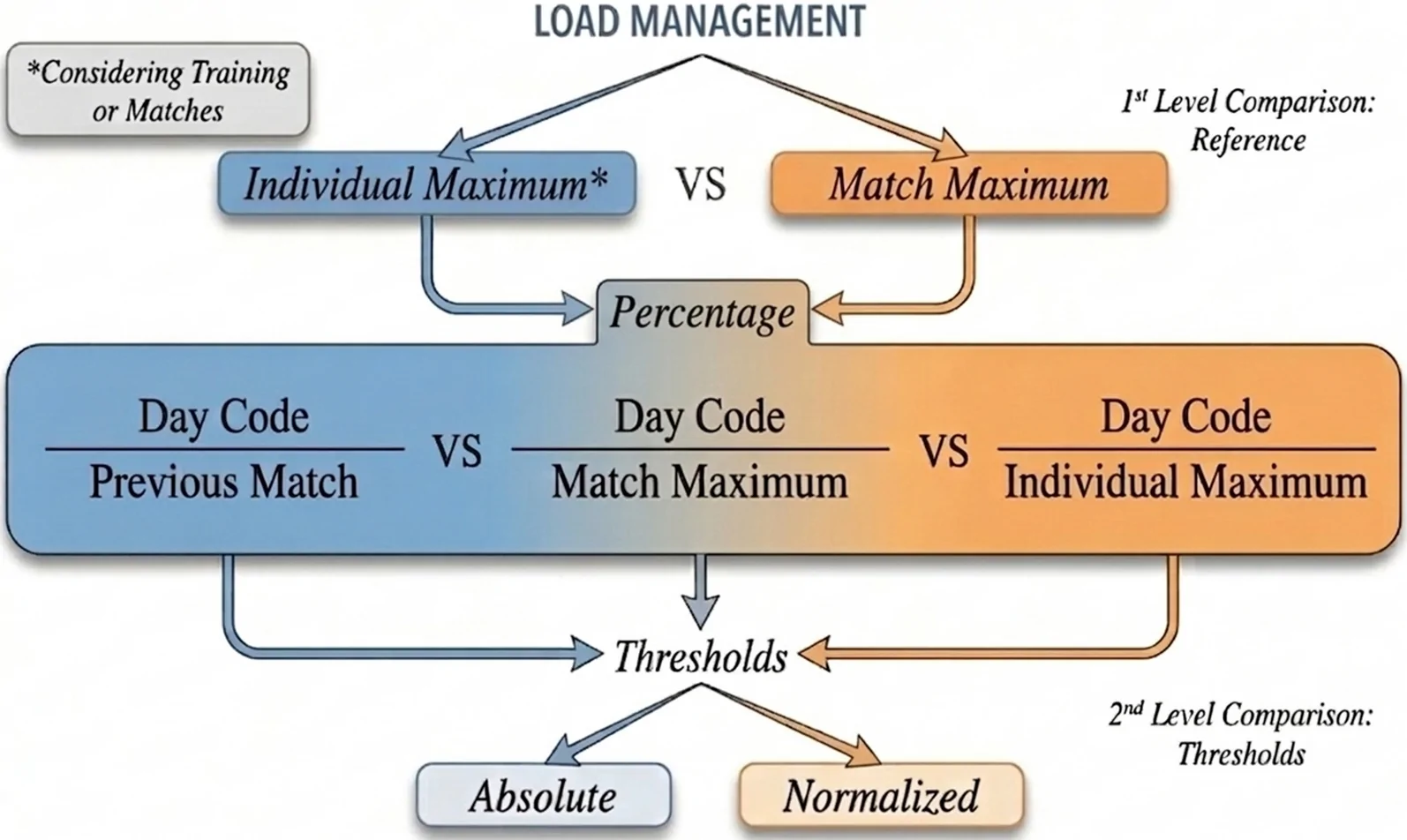
Conceptual framework for load management analysis. Training and match loads were contextualized using three exposure-based reference points: the previous match, match maximum, and individual maximum, with the latter representing the highest external load observed across training and match contexts. Percentages were calculated to compare load (day code) relative to (1) the previous match, (2) the match maximum, and (3) the individual maximum. These comparisons represent the first-level reference framework. Subsequently, absolute and normalized threshold approaches were conceptually contrasted as a second reference level for interpreting training and match locomotor external loads within the microcycle

Research indicates that, on average, medium- and high-intensity ACC and DEC during training were similar to match values [8], although with high variability (39–90%). This variability further amplifies discrepancies between absolute and normalized load metrics, with relevant methodological and practical considerations. Accordingly, practitioners could be underestimating or misrepresenting the external load performed by each player. To address this, we propose that external load can be contextualized relative to appropriate individual reference values (Fig. 2), as a conceptual approach to support a more player-specific interpretation of match locomotor external load and training prescription and monitoring.

**Fig. 3:**
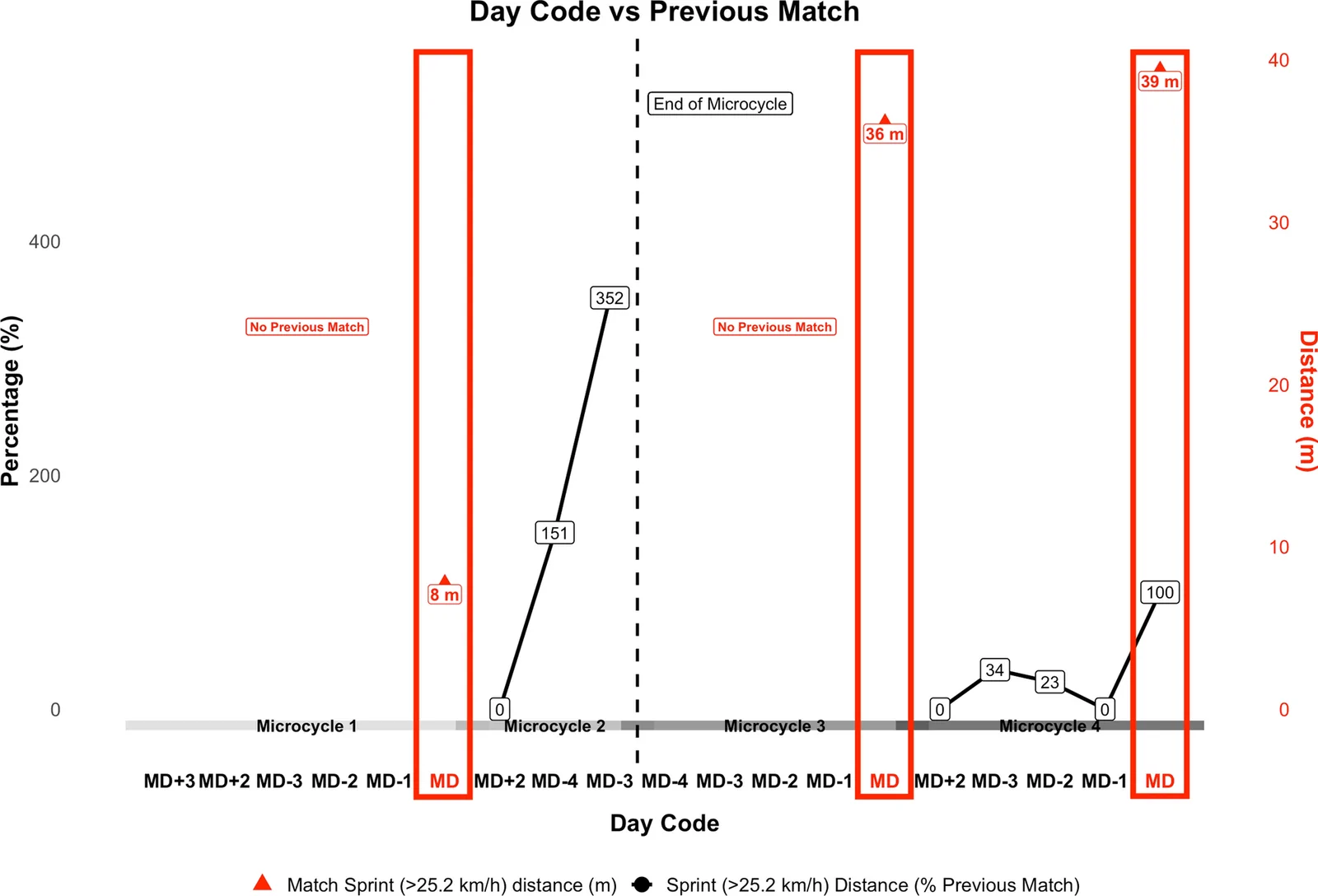
Percentage between training and the previous match load across microcycles for absolute sprint (Sprint_ABS_, distance covered ≥ 25.2 km/h). Values represent the proportion of training load performed on each microcycle day (MD−4, MD−3, MD−2, MD−1) relative to the locomotor external load of the preceding match. The “No Previous Match” condition indicates that, within that specific microcycle, no comparison with prior match locomotor external load is possible due to the absence of a preceding competitive fixture. The dashed vertical line denotes the point at which microcycle 3 concluded. Data are derived from the anonymized unpublished monitoring dataset described in Sect. "Methodological framework" and are presented solely for illustrative purposes; they are not intended as empirical validation of the proposed framework. MD, match day

**Fig. 4:**
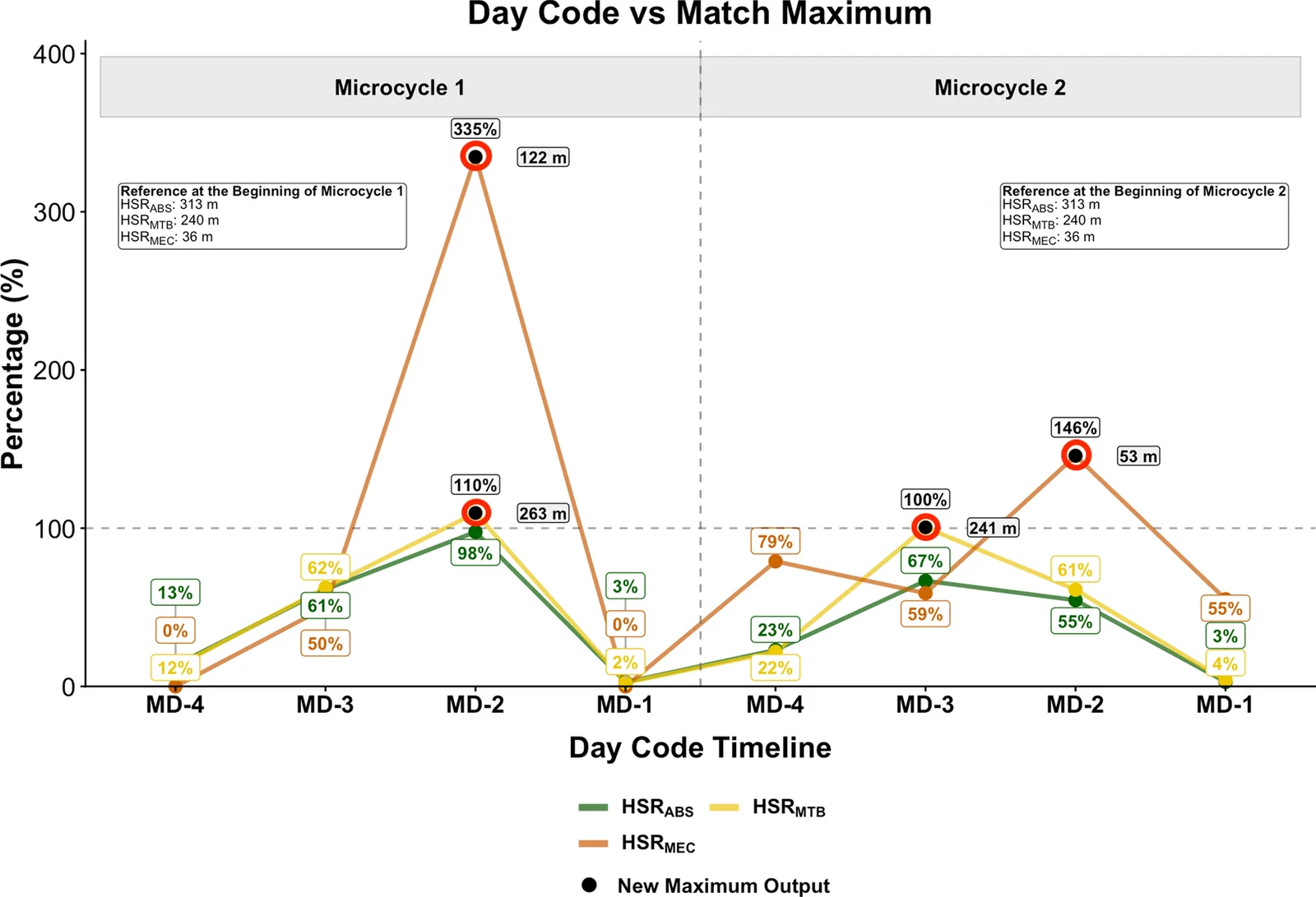
Comparison of training-session distances in HSR_ABS_ (absolute high-speed running; distance accumulated between 19.8 and 25.2 km/h), HSR_MTB_, metabolic high-speed running; distance accumulated at ≥60–<75% of maximum speed), and HSR_MEC (_mechanical high-speed running; distance accumulated at ≥75–<90% of maximum speed), normalized to the peak values recorded during competitive match play, across two consecutive microcycles. The dashed horizontal line represents 100% of the maximal match-derived load. Data are derived from the anonymized unpublished monitoring dataset described in Sect. "Methodological framework" and are presented solely for illustrative purposes; they are not intended as empirical validation of the proposed framework

**Fig. 5:**
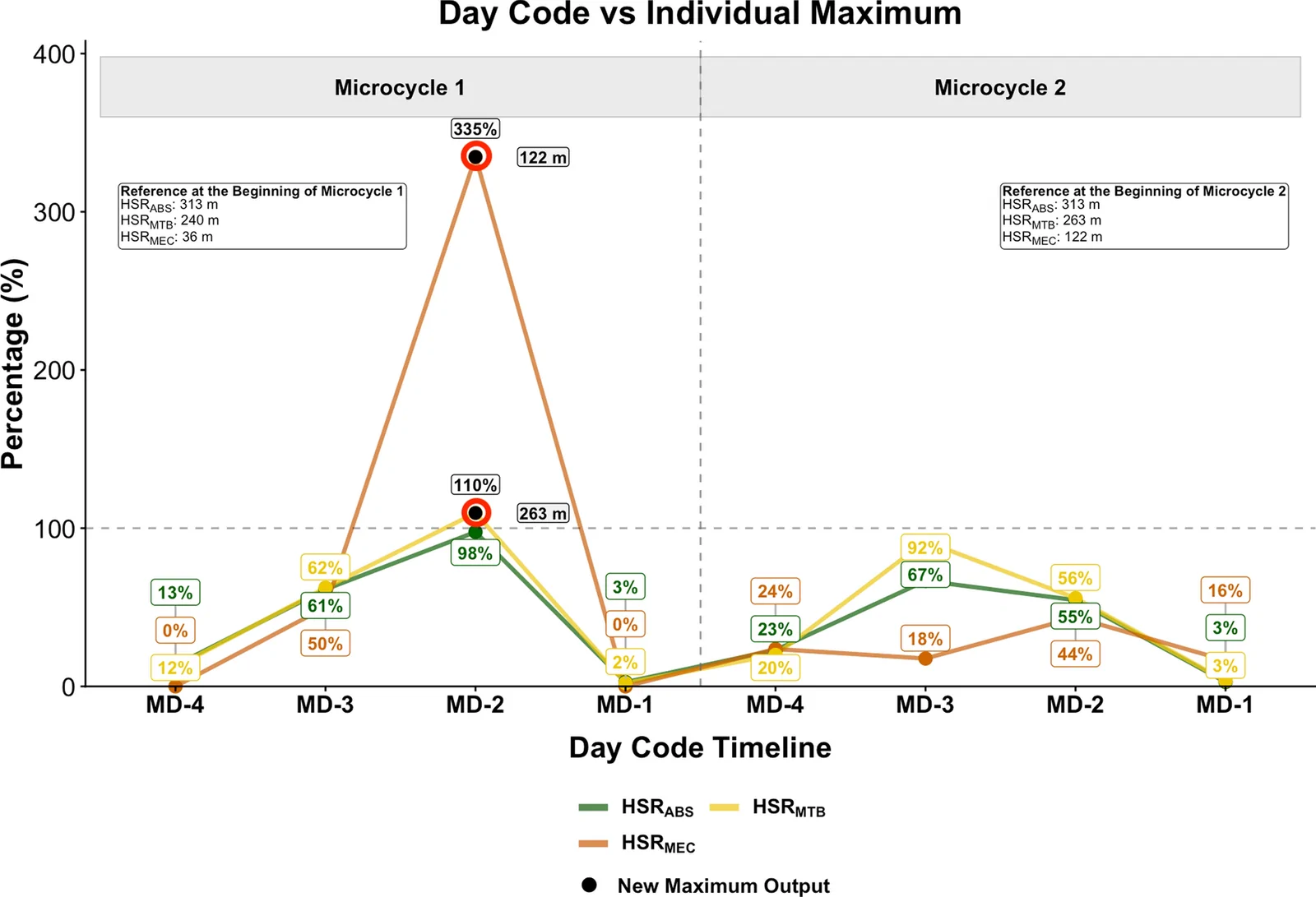
Comparison of training-session distances in HSR_ABS_ (absolute high-speed running; distance accumulated between 19.8 and 25.2 km/h), HSR_MTB_ (metabolic high-speed running; distance accumulated at ≥60–<75% of maximum speed), and HSR_MEC_ (mechanical high-speed running; distance accumulated at ≥75–<90% of maximum speed) normalized to the peak values (distances) recorded during all activities in which the player participated within the analyzed microcycles. Data are derived from the anonymized unpublished monitoring dataset described in Sect. "Methodological framework" and are presented solely for illustrative purposes; they are not intended as empirical validation of the proposed framework

To illustrate the conceptual differences among the proposed reference approaches, anonymized Global Navigation Satellite System (GNSS)-derived external load data from a high-level football player were obtained from routine performance monitoring conducted during a season. External training load was monitored using a portable 10 Hz GNSS device (CatapultVector S7, Catapult Sports, Melbourne, Australia). These data were used solely to construct Figs. 3, 4, 5, and 7 and to demonstrate how the interpretation of the same training exposures may change according to the selected reference value. No inferential analyses were performed, and these illustrative data are not intended to provide empirical validation of the proposed conceptual framework.

### Day Code vs Previous Match Load

The magnitude of load observed in a single match is inherently influenced by contextual and situational factors, meaning that match-derived values reflect context-dependent external load rather than the highest locomotor output that the player may be capable of producing. Indeed previous findings demonstrate that match load is modulated by game-related variables, including scoring status [28–31]. For instance, entering the second half with a substantial lead may promote a more conservative game-management strategy, reflected in reduced use of build-up, sustained attacking threat, crossing, and high-pressure behaviors [32]. A favorable match status has also been associated with lower high-intensity activity, while reductions in running intensity and explosive-load measures from the first to the second half have been observed across several scoreline scenarios [33, 34]. Conversely, when trailing, teams may attempt to regain possession in more advanced areas and perform more intense pressing-related actions [35, 36] while increasing build-up, sustained attacking threat, crossing, and fast-tempo play [32].

Further reinforcing this rationale, research has shown that the quality of opposition significantly influences match external load [31, 37]. Specifically, a positive association has been observed between the external load recorded during match-play and the competitive standard of the opposing team. Matches against higher-quality opponents tend to elicit greater locomotor external load, whereas lower-quality opposition may not sufficiently stimulate maximal external load [38].

Taken together, these findings suggest that using the previous match as a fixed reference for training prescription could introduce substantial bias, as the derived load is contingent upon contextual determinants rather than representing a stable and reproducible indicator of a player’s highest observed external load (Fig. 3).

Importantly, the interpretation of locomotor load should consider the specific variable being examined, as equivalent relative values across different metrics do not necessarily represent equivalent external load characteristics. For example, a high relative total distance represents a different locomotor exposure from a small number of high-intensity accelerations or decelerations. Moreover, match play encompasses additional load components (e.g., impacts and collisions) that may contribute to post-match fatigue, meaning that relatively low values for a given locomotor metric do not necessarily indicate a low overall match load.

Match-induced fatigue can persist for more than 72 h and is modulated by factors, such as playing standard [7, 39], prior locomotor outputs [19], and match location [40, 41]. Accordingly, practitioners commonly assess acute (0–24 h) and residual (> 24 h) fatigue to inform training prescription. Although metrics such as HSR_ABS_ have been associated with biochemical and neuromuscular markers of fatigue [42], these relationships are typically derived from absolute values and may not account for inter-individual variability in internal load responses. Therefore, two players may record the same external load when quantified using the same absolute threshold while exhibiting substantially different internal load responses. Moreover, the same absolute external load may represent different locomotor intensities according to each player’s individual physical load profile [15].

This limitation is further exacerbated in the absence of prior match data, such as at the beginning of the season or in players who are frequently non-starters. In these cases, the lack of a valid reference precludes the establishment of player-specific benchmarks, limiting practitioners’ ability to determine whether players are being exposed to loads that approximate their maximum output. This has practical implications, as it constrains decision-making related to readiness, rotation, and return to play, potentially increasing the risk of underpreparation or maladaptation.

### Day Code vs Maximum Match Load

The limitations inherent to the use of previous match load as a reference also extend to approaches in which the daily load is expressed as a percentage of the maximum match load (e.g., day load/reference load) (Fig. 4).

Although, in theory, the discrepancy between match-derived load and the player’s highest observed load may be reduced when the maximum match value is used, this approach remains conceptually limited. Specifically, using the maximum match load as the reference assumes that players are able to reach their highest locomotor outputs during competitive match play. However, this assumption is not unequivocally supported by the literature. Evidence indicates that players may reach higher load magnitudes in non-competitive scenarios, such as structured training drills [27] or testing protocols [26], where task constraints are deliberately manipulated to target specific locomotor outputs and intensities. Indeed, as shown in Fig. 4, specifically during microcycle 1, higher distances were observed in HSR_MTB_ and HSR_MEC_ during MD−2 sessions compared to the match maximum.

Moreover, match external load is strongly influenced by contextual variables, including opposition quality [31, 37, 38]. Lower-level competitions are likely to impose lower external load compared with higher-tier competitions, a notion partially supported by studies reporting significant differences in locomotor outputs across competitive standards [43]. Consequently, even when the maximum match load is used as a reference, the absolute magnitude of that value may not accurately represent the player’s highest observed locomotor output. If maximum match load were assumed to represent the upper limit of each player’s locomotor output, it would be difficult to explain how athletes transitioning from lower-tier competitions are subsequently able to tolerate the higher external loads observed at elite levels. This limitation becomes even more evident in the case of non-starters and non-selected players. Non-starters who have never completed a full match are likely to accumulate lower absolute match loads compared with a hypothetical full-match exposure [44, 45]. This discrepancy becomes even more pronounced for players who have participated only for limited minutes or have not been used at all. As such, the maximum match load identified for non-starters is unlikely to approximate their highest locomotor load to the same extent as it might for regular starters. For non-selected players, the absence of match exposure eliminates the possibility of establishing any match-derived reference value.

The limitations associated with using maximum match load as the reference for external load prescription are also evident in return-to-play (RTP) contexts. In applied settings, players undergoing rehabilitation are progressively exposed to training loads based on their pre-injury match-derived external load values [46, 47]. Conceptually, the final phase of an RTP process is expected to ensure that the player can tolerate, at minimum, the highest external load previously registered during competitive match play. However, this rationale remains methodologically limited. As previously discussed, the maximum external load recorded during a match does not necessarily represent the player’s highest observed external load across football activities. Match outputs are influenced by contextual variables (e.g., tactical demands, opposition quality, playing time, and match result), and higher locomotor loads may subsequently be observed during training or competition. Consequently, basing RTP progression on match-derived maximum values may result in an underestimation of the load threshold that the player must ultimately tolerate upon full reintegration into unrestricted training and competition. From a risk-management perspective, this approach may be insufficient to ensure adequate physical preparedness, particularly considering the stochastic and high-intensity nature of match play. Accordingly, achieving a previous match-derived maximum during rehabilitation does not necessarily indicate that the player has been exposed to the highest locomotor loads that may occur following return to unrestricted football participation. Therefore, in RTP processes, match-load-derived references should be interpreted cautiously and, where possible, complemented by player-specific maximum exposure- and characteristic-based reference values that may better represent maximum or near-maximal locomotor outputs across training and competition.

It should also be acknowledged that match locomotor external load can be characterized using different temporal approaches, including full-match values, individual match periods, and peak-intensity rolling windows, which may provide complementary information regarding the temporal distribution of locomotor activity. However, these approaches remain conceptually distinct from the selection of the reference used to normalize external load. Collectively, these considerations suggest that the use of maximum match load remains a fundamentally reactive approach to load monitoring. Coaches must wait for competitive exposure to observe peak values, meaning that load prescription is based on context-dependent match outputs rather than on the player’s highest observed locomotor load. This highlights the need to distinguish exposure-based references derived from broader training and match observations from characteristic-based references obtained under conditions specifically designed to assess individual characteristics, such as maximum speed.

### Day Code vs Maximum Individual Load

As discussed above, players may not necessarily express their highest locomotor outputs during competitive matches due to the variability introduced by contextual and situational factors. Higher load magnitudes may be observed in comparatively controlled environments, such as structured training drills or dedicated testing sessions, where task constraints are deliberately manipulated to target specific locomotor outputs and intensities (Fig. 5). Nevertheless, the interpretation of normalized cumulative locomotor exposure should consider the context in which such exposure occurs, particularly when preceding activity and accumulated fatigue differ.

Beyond the conceptual consideration associated with using maximal match load as a reference for external load prescription, an additional limitation concerns the frequency of observations. The number of match exposures across a season is substantially lower than the number of training sessions. Given the greater volume of training observations, there is a higher probability of capturing a broader range of locomotor outputs, including the player’s highest observed external load values. Training and testing environments provide a relatively controlled framework in which coaches intentionally manipulate task constraints to target specific locomotor outputs and intensities, thereby providing more controlled physiological and mechanical stimuli. Importantly, individual reference values should not necessarily be considered static throughout the season, as both maximum observed loads and individual characteristics may change over time. Accordingly, maximum observed loads should be continuously monitored, while periodic characteristics may provide more representative reference values than relying exclusively on pre-season testing.

**Fig. 6:**
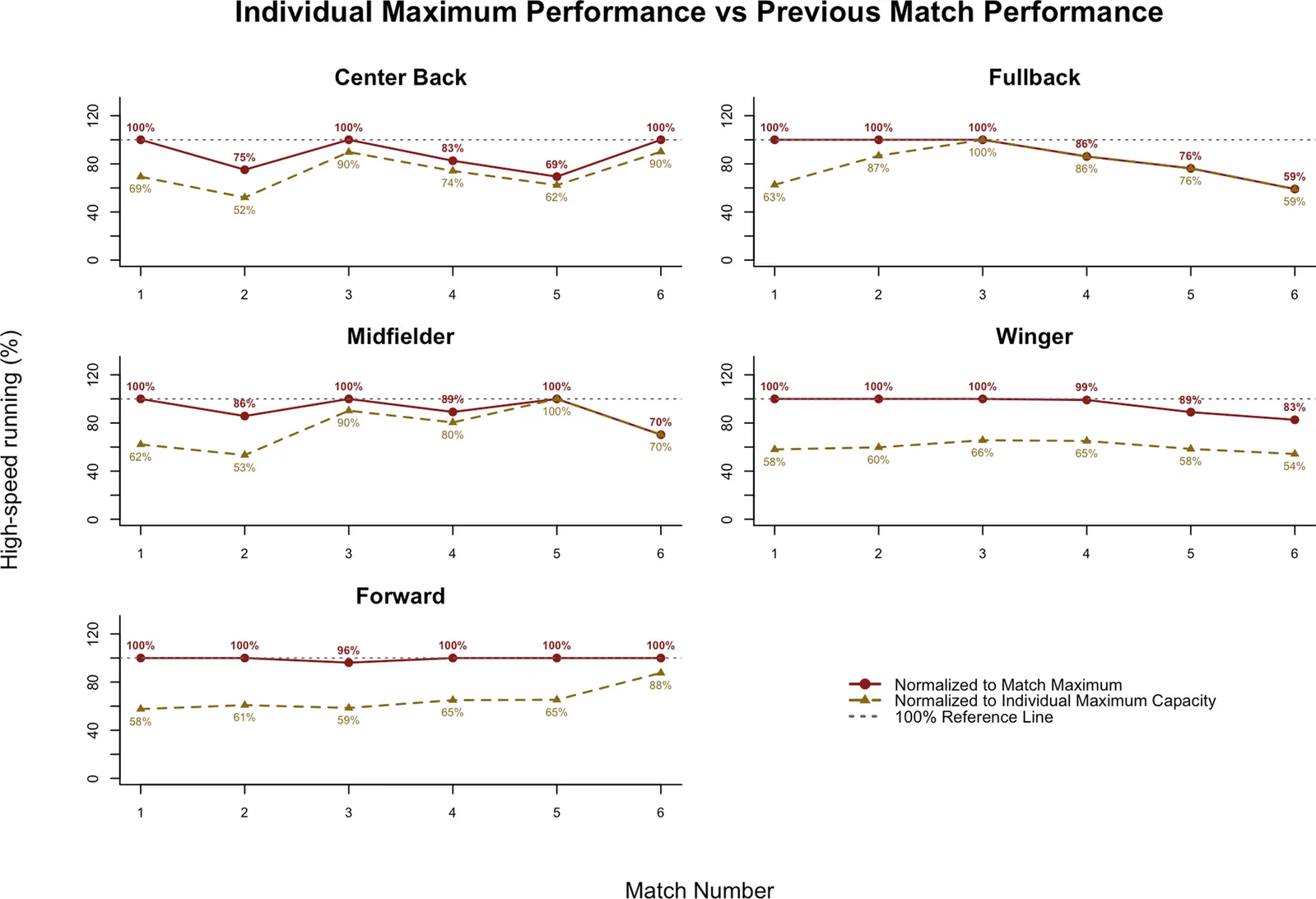
Schematic representation of the differences regarding the use of the match load or maximum average load for each playing position as references for monitoring the external load produced across the first six matches. Random values were plotted based on averages, standard deviations, coefficients of variation, and respective 95% confidence intervals for each position. Adapted from Gregson et al. (2010)

Regarding training, it has been reported that higher acceleration and deceleration loads are often observed during MD−4 sessions [48, 49] compared with other microcycle day codes, whereas greater sprint distances may be recorded during MD−2 sessions [50]. These systematic manipulations reflect deliberate programming decisions aimed at overloading specific locomotor metrics. Indirectly, this practice challenges the validity of using match-derived loads (either previous match load or maximal match load) as the primary reference for training prescription. According to the principle of specificity, match play would represent the most ecologically valid stimulus to which players are exposed. However, scientific evidence and applied practice suggest that coaches do not replicate full match external load in every session of a microcycle [22], as doing so would likely result in excessive cumulative fatigue. Instead, a variety of task formats, including small-sided and large-sided games, alongside other training drills, are implemented with the objective of reproducing specific percentages of match external load [51]. Nevertheless, if match load does not accurately represent the player's highest observed external load, training prescription based exclusively on match-derived references may constrain the locomotor stimulus prescribed. In contrast, prescribing exercise intensity relative to individual characteristic-based references allows training to target the development of physical capacities rather than simply reproducing the highest external load observed during competition (Fig. 6).

Given that exercise-induced adaptations are fundamentally mediated by training intensity the systematic exposure to appropriate magnitudes of intensity and volume is essential to elicit meaningful training responses. Match-derived loads may not fully represent the intensities required to target specific physical qualities. Therefore, we propose a proactive programming framework that integrates the highest individual external load values observed across training and competition with appropriate player-specific references. Such an approach may provide a more player-specific basis for load prescription without assuming that maximum observed external load and the true maximum of the player represent the same construct.

## Normalized vs Absolute Thresholds to Analyze Match Locomotor Performance

Previous research shows that the quantification of external load varies substantially depending on the use of absolute or normalized thresholds to characterize locomotor intensities [15, 16, 18]. Absolute thresholds may systematically under- or overestimate individual load. For instance, common ACC and DEC absolute thresholds (> 3 m/s^2^ and < −3 m/s^2^), are considerably lower than reported maximum values (ACC_max_ ≈ +67%; DEC_max_ ≈ − 133) [17]. Similarly, Sprint_ABS_ may correspond to approximately 72% of the maximum speed produced by professional football players [16], thereby failing to reflect near-maximal sprinting.

Given the constrained and opponent-driven nature of match play, players may not consistently reach maximal intensities during competition [18]. Evidence indicates that peak locomotor outputs may not be fully expressed in competition, due to contextual and tactical constraints [52]. Although previous studies have not explicitly compared match and more controlled training contexts, their findings suggest that training exercises in which football-specific contextual constraints are reduced may provide more appropriate conditions for players to express targeted physical capacities or locomotor outputs. For example, it has been shown that a subset of players is unable to reach speeds exceeding 95% of their maximum speed during match play [18], while maximum speeds could be reached during 40-m straight line sprint drill [18, 27]. Similarly, greater deceleration performance has been reported under standardized testing (e.g., 5–0–5 change-of-direction tests conducted in the pre-season) compared with in-season match and training observations [26]. Consequently, using match-derived locomotor outputs as a reference for training prescription may lead practitioners to underestimate players' locomotor load and inadvertently understimulate locomotor-related qualities. Therefore, characteristic-based references should be derived from reliable estimates of each player’s maximum, preferably obtained from consistent maximal observations under appropriate assessment conditions rather than from a single isolated value that may be influenced by measurement error.

**Fig. 7:**
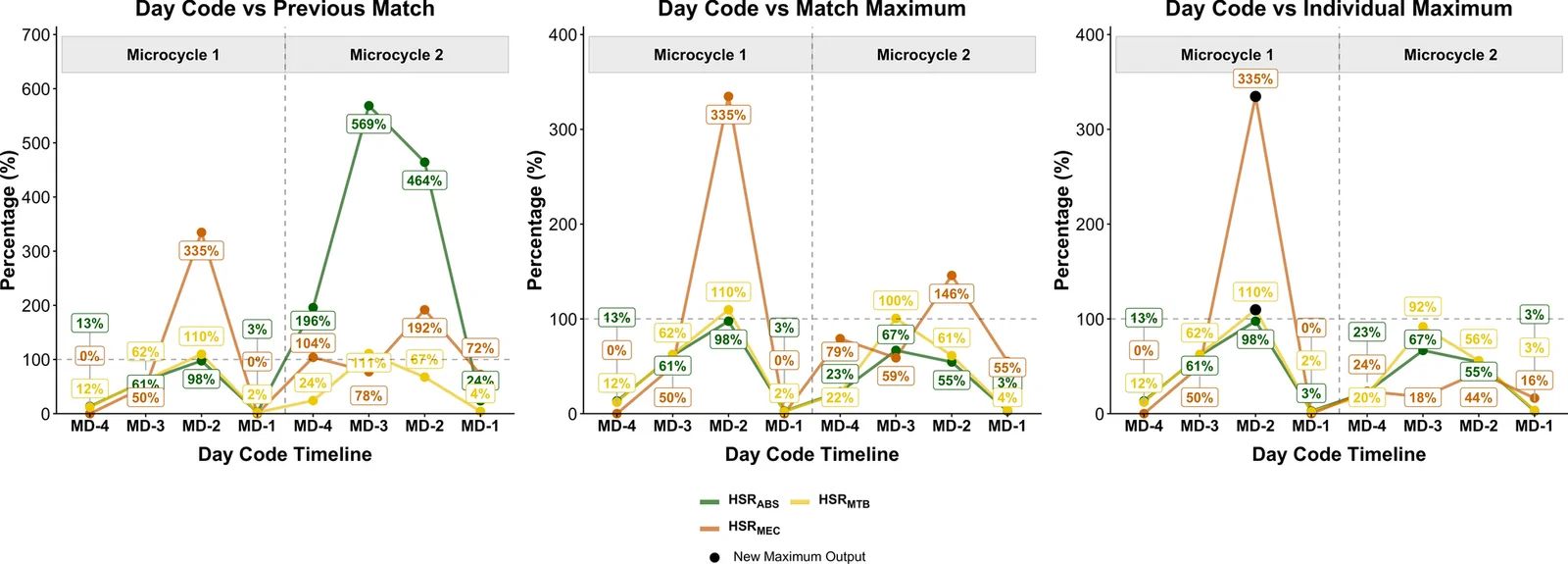
Comparison of training-session distances in HSR_ABS_ (absolute high-speed running; distance accumulated between 19.8 and 25.2 km/h), HSR_MTB_ (metabolic high-speed running; distance accumulated at ≥60–<75% of maximum speed), and HSR_MEC_ (mechanical high-speed running; distance accumulated at ≥75–<90% of maximum speed) normalized to the peak values (distances) recorded during (A) the previous match, (B) the match maximum considering all matches played up to that time, and (C) all activities in which the player participated within the analyzed microcycles. Note that *y*-axis ranges differ between panels to accommodate differences in the magnitude of normalized values. Data are derived from the anonymized unpublished monitoring dataset described in Sect. "Methodological framework" and are presented solely for illustrative purposes; they are not intended as empirical validation of the proposed framework

If such match-derived load exposure patterns are replicated chronically across the season, players may become underprepared to tolerate the mechanical and neuromuscular demands imposed when match contexts unexpectedly elicit higher running intensities. This under-exposure could increase vulnerability to fatigue or injury during future competition [53], particularly when players are required to perform intensities that have not been regularly attained in preceding training or match contexts [21]. Therefore, reliance on match load as a reference for sprint training prescription may contribute to inadequate preparation for competition and suboptimal training adaptation, as absolute threshold-based locomotor metrics often overestimate individual high-intensity match locomotor outputs. In other sporting contexts, such as athletics, practitioners commonly use an athlete’s “personal best” or “seasonal best” performances as reference standards to monitor progression throughout the competitive year. These benchmarks provide a player-specific performance anchor against which current outputs can be systematically evaluated. A comparable framework is proposed herein for football, whereby players’ locomotor performance is interpreted relative to their individual “personal best” loads. This approach enables the comparison of current match or training outputs against the athlete’s highest recorded load, which may not necessarily be attained during competitive match play (Fig. 7). Consequently, such a player-specific reference with a normalized maximum-load threshold system may offer a more sensitive and performance-oriented method for tracking development and contextualizing external load exposure. Ultimately, long-term athletic development may benefit from focusing on improving individual physical capacities rather than merely reproducing external loads derived from a context-dependent match maximum.

## Conclusions

A more player-specific and methodologically explicit framework may improve the interpretation and prescription of external load in football. Normalizing locomotor metrics using player-specific characteristic-based and/or exposure-based individual references represents one potential approach toward a more player-specific interpretation of external load. Characteristic-based references may be used to characterize locomotor intensity relative to the player’s individual characteristics, whereas exposure-based references contextualize cumulative locomotor load relative to the highest values previously observed across training and competition. Importantly, the framework proposed is conceptual and should not be interpreted as providing empirically validated thresholds or decision rules. Longitudinal research is required to determine its validity and practical utility in football practice.

Further research is warranted to determine whether fatigue responses align more closely with the expected magnitude and direction when external loads are expressed using normalized rather than absolute thresholds. Although previous studies have reported associations between locomotor metrics and post-match fatigue persisting beyond 48 h [42, 54], these findings do not account for the relative intensity of efforts in relation to individual maximum.

From a practical perspective, the proposed framework may provide a structure for exploring how normalized volumes could inform the interpretation of fatigue-related locomotor metrics. Such interpretations should also integrate contextual, cognitive, and emotional stressors, as well as additional external load components that are more prevalent during match play (e.g., impacts and collisions). Within this conceptual framework, values approaching or exceeding maximum normalized volumes could indicate the need for enhanced recovery strategies, whereas sub-maximal volume exposure (depending on its magnitude) could represent an opportunity to apply targeted training stimuli to promote specific physical adaptations, rather than uniformly prescribing recovery across the squad. However, these interpretations remain hypothetical and require empirical validation before they can be translated into prescriptive decision rules. This normalized approach contrasts with the prevailing match-centric paradigm, where load management is commonly driven by the previous and upcoming matches. Given the time course of physiological adaptations, meaningful improvements in physical capacity are unlikely to occur within a single microcycle, creating a temporal mismatch between short-term performance priorities and longer-term physical development.

Collectively, the reliance on match load as the primary reference for external load prescription presents substantial limitations. Due to the inherently stochastic and context-dependent nature of match play, match-derived metrics may underestimate both the player’s highest external load and the locomotor intensity performed. This misrepresentation could contribute to suboptimal load distribution across the microcycle and influence subsequent training prescription decisions. In turn, this may affect the extent to which players are exposed to the locomotor volumes and intensities targeted within the training process. In contrast, normalization of locomotor loads using appropriate reference values may provide a more stable, sensitive and performance-oriented reference framework, which could support a more precise external load prescription and monitoring. Future research should extend the present framework using longitudinal data from professional football practice to determine the extent to which these reference approaches produce meaningful differences in training prescription and load-monitoring decisions.

## Data Availability

The unpublished monitoring data used to construct the illustrative Figs. 3, 4, 5, and 7 are available from the corresponding author upon reasonable request. These data were used solely for illustrative purposes and were not analyzed for hypothesis testing or empirical validation of the proposed framework.

## Abbreviations

ACC: Acceleration
ACC_ABS_: Absolute acceleration
ACC_max_: Maximum acceleration
CV: Coefficient of variation
DEC: Deceleration
DEC_ABS_: Absolute deceleration
DEC_max_: Maximum deceleration
GNSS: Global Navigation Satellite System
HSR_ABS_: Absolute high-speed running
HSR_MEC_: Mechanical high-speed running
HSR_MTB_: Metabolic high-speed running
MD: Match day
MD±: Match day minus/plus
RTP: Return to play
Sprint_ABS_: Absolute sprint distance
TD: Total distance
